# The ON Crossover Circuitry Shapes Spatiotemporal Profile in the Center and Surround of Mouse OFF Retinal Ganglion Cells

**DOI:** 10.3389/fncir.2016.00106

**Published:** 2016-12-22

**Authors:** Jasdeep Sabharwal, Robert L. Seilheimer, Cameron S. Cowan, Samuel M. Wu

**Affiliations:** ^1^Medical Scientist Training Program, Baylor College of MedicineHouston, TX, USA; ^2^Department of Neuroscience, Baylor College of MedicineHouston, TX, USA; ^3^Department of Ophthalmology, Baylor College of MedicineHouston, TX, USA

**Keywords:** mouse retina, ganglion cell, retinal circuitry, antagonistic surround, amacrine cell, crossover circuit, L-AP4, spatiotemporal processing

## Abstract

Retinal ganglion cells (RGCs) are often grouped based on their functional properties. Many of these functional properties, such as receptive field (RF) size, are driven by specific retinal circuits. In this report, we determined the role of the ON bipolar cell (BC) mediated crossover circuitry in shaping the center and surround of OFF RGCs. We recorded from a large population of mouse RGCs using a multielectrode array (MEA) while pharmacologically removing the ON BC-mediated crossover circuit. OFF sustained and transient responses to whole field stimuli are lost under scotopic conditions, but maintained under photopic conditions. Though photopic light responses were grossly maintained, we found that photopic light response properties were altered. Using linear RF mapping, we found a significant reduction in the antagonistic surround and a decrease in size of the RF center. Using a novel approach to separate the distinct temporal filters present in the RF center, we see that the crossover pathway contributes specifically to the sluggish antagonistic filter in the center. These results provide new insight into the role of crossover pathways in driving RGCs and also demonstrate that the distinct inputs driving the RF center can be isolated and assayed by RGC activity.

## Introduction

The retina is thought to process the visual scene by using up to 30 parallel neural circuits which signal to downstream visual pathways via distinct retinal ganglion cells (RGCs) subtypes (Roska and Werblin, 2001; Baden et al., 2016). These RGC subtypes have different space-time properties that arise due to the specific makeup of their upstream circuitry (Peichl and Wässle, 1983; Kolb, 1995; Field et al., 2007; Völgyi et al., 2009). Mouse has been a common model for studying the relation of circuit and function because there are many tools to dissect circuit components (Sinclair et al., 2004; Coombs et al., 2006; Abd-El-Barr et al., 2009). Studies have investigated the roles of specific upstream circuits in RGC sensitivity (Völgyi et al., 2004; Cowan et al., 2016a), but there has not been a consensus on the role of specific circuits in complex features of RGCs such as center-surround receptive fields (RFs) and space-time tuning.

While ON and OFF RGCs are primarily driven by ON and OFF bipolar cells (BCs), respectively, it has been shown that there is crossover between these two pathways (Werblin, 2010). For example, ON-BCs interact with the OFF pathway via amacrine cell networks (ON crossover; Wässle et al., 1986; Molnar and Werblin, 2007). This pathway can be isolated by recording from OFF pathway components while using L-2-amino-4-phosphonobutyrate (L-AP4), an mGluR6 agonist that specifically blocks photoreceptor to ON-BC signaling (Shiells et al., 1981; Slaughter and Miller, 1981; Schiller, 1982). The ON crossover pathway has been shown to contribute to multiple aspects of OFF pathway function by feedback to the BC (Molnar and Werblin, 2007; Rosa et al., 2016) and feedforward to the ganglion cell (Manookin et al., 2008).

Our lab recently developed a novel approach to analyze the linear space-time RF of RGCs. This approach allows us to divide the space-time RF into several components. These components not only capture classic RF elements like the center and antagonistic surround, but also others, such as the sluggish antagonistic center, which were previously difficult to characterize (Cowan et al., 2016b). By pairing this model with circuit dissection, we can determine how specific circuits alter RGC space-time tuning in the center and surround.

Here we studied the role of ON crossover circuits by utilizing L-AP4 while recording from a population of OFF RGCs on a Multielectrode array (MEA). There is broad preservation of low photopic responsivity, but loss of scotopic responsivity. By using linear analysis, we found that ON crossover circuits contribute to both the center and surround RFs of OFF RGCs under photopic conditions. In the center, the ON crossover circuits also slow the OFF RGC response kinetics. Together these results provide evidence for multiple roles of ON crossover pathways in shaping OFF RGC spatiotemporal responses.

## Materials and Methods

### Ethical Approval

Mice were cared for and handled following approved protocols from the Animal Care and Use Committee of Baylor College of Medicine and in compliance with the National Institutes of Health guidelines for the care and use of experimental animals. All mice were euthanized by cervical dislocation while under a surgical plane of anesthesia.

### Multielectrode Array (MEA) Recording

Nine male C57Bl/6J mice were kept on a regular light/dark cycle and experiments were performed diurnally at 3–4 months of age. Mice were dark adapted for at least 90 min prior to euthanasia. Retinal electrophysiology was carried out as indicated in our previous publications (Cowan et al., 2016a,b). In brief, eyes were removed under infrared illumination using night vision scopes (Nitemare, BE Meyers, Oregon) and whole-mount retinas were placed onto a MEA, ganglion cell side down. Recordings were made primarily from central retina. The MEA (MEA-60, Multichannel Systems, Tübingen Germany) had 60 electrodes spaced 100 μm apart, each with a diameter of 10 μm. Ganglion cell action potentials were recorded at 20 KHz and pre-filtered with a 0.1 Hz high-pass hardware filter.

**Figure 1:**
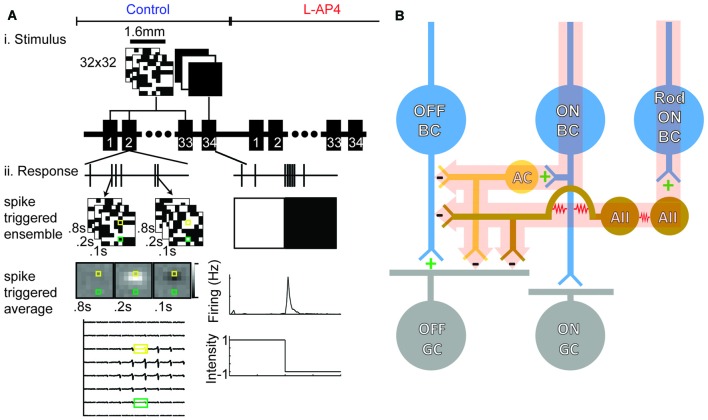
**Retinal ganglion cell (RGC) spikes are used to determine space-time receptive fields (RFs) and functional properties. (A)** White noise checkerboards were presented for 90 min (166 s trials, 33 presentations). Sample analysis for one experiment is shown: peri-spike stimuli were found and averaged to generate a spike-triggered average (STA). The bottom left panel shows the STA for an example OFF cell. Whole field light steps are presented for 4 min (4 s light offset, 4 s light onset, 30 presentations). Firing rates were averaged across the 30 trials to generate a trace as shown at the bottom right. This is an example of an OFF-transient GC. **(B)** Simplified circuit diagram to illustrate which circuits are inactivated with L-2-amino-4-phosphonobutyrate (L-AP4). The ON-bipolar cell (BC) contributes the major input to ON-GCs but also provides crossover input to the OFF pathway via feedback to OFF-BCs and feedforward to OFF-GCs (highlighted pathway). Addition of L-AP4 blocks all ON pathway signaling.

The retina was kept at 35.6°C and perfused with carboxygenated (95% O_2_, 5% CO_2_) recording solution (in mM: NaCl, 124; KCl, 2.5; CaCl_2_, 2; MgCl_2_, 2; NaH_2_PO_4_, 1.25; NaHCO_3_, 26; and glucose, 22 at pH 7.35; Tian and Copenhagen, 2003). Experiments were first performed in the standard recording solution, and then with 20 μM L-AP4 mixed into the solution (Figure 1A). In some experiments, additional recordings were performed in standard recording solution after the drug was washed out.

### Spike Sorting

Potential spikes were identified from each recording as signals greater than 3 standard deviations from baseline. These were sorted using a clustering algorithm (Kadir et al., 2014), based on key features for the waveform of each spike. These features included spike amplitude, spike shape and the electrical footprint. The electrical footprint was identified as the activity on every other channel during each potential spike, thus outlining a shape of the axon for each cell. Each potential unit from this clustering method was assessed for contamination based on a ratio of the firing rate within the refractory period to the overall firing rate of the cell. These methods allowed us to assign spikes before and after L-AP4 to the same cell. There is no significant difference between a unit’s average waveform shape of spikes or its electrical footprint before and after L-AP4 (data not shown).

### Light Calibration

Similar to our previous report (Cowan et al., 2016b) and those of others (Pandarinath et al., 2010b), the ambient white light level during an experiment was measured as wavelength specific irradiance (E(λ), in microwatts cm^−2^) in the plane of the preparation (Thor Labs, S170C and Edmund Optics, SpectraRad). The mean ambient photopic light level was 757.9 R*/rod/s and the monitor had a contrast of −1 to 1. Neutral density filters were used to create three log unit attenuation, creating an ambient scotopic light level of 0.8 R*/rod/s. Stimuli were projected as an optically reduced image from a computer monitor which presented light from the visible spectrum (Dell, SXGA-JF311-5100). A beam splitter was used to present the image from the computer monitor from below the MEA.

### Whole Field Light Stimulation

Whole field light steps were 30 repeated trials of 4 s of a black screen followed by 4 s of a white screen. ON/OFF and sustained/transience was determined as described in other reports (Della Santina et al., 2013; Cowan et al., 2016a). The number of spikes occurring during light onset and offset were summed and used to compute the ON-OFF index as shown in equation 1.

(1) $$\text{ON}/\text{OFFIndex}\;=\;\frac{ON_{\text{spikes}}-OFF_{\text{spikes}}}{ON_{\text{spikes}}+OFF_{\text{spikes}}}$$

### White Noise Receptive Field Mapping

RFs were mapped using random binary white noise checkerboards presented at 15 Hz. Each square in the checkerboard was either black or white and 50 μm on a side. The stimulus was created and presented with PsychToolbox (Brainard, 1997; Pelli, 1997). Reverse correlation was used to compute a space-time spike-triggered average (STA; Meister et al., 1994; Chichilnisky, 2001). A depiction of reverse correlation is shown in Figure 1A. Given the 15 Hz stimulation, we were limited to responses up to 7.5 Hz. Based on the corner frequencies of our temporal filters and previous studies carried out under similar light levels the RGC responses should fall well below this (Pandarinath et al., 2010b).

**Figure 2:**
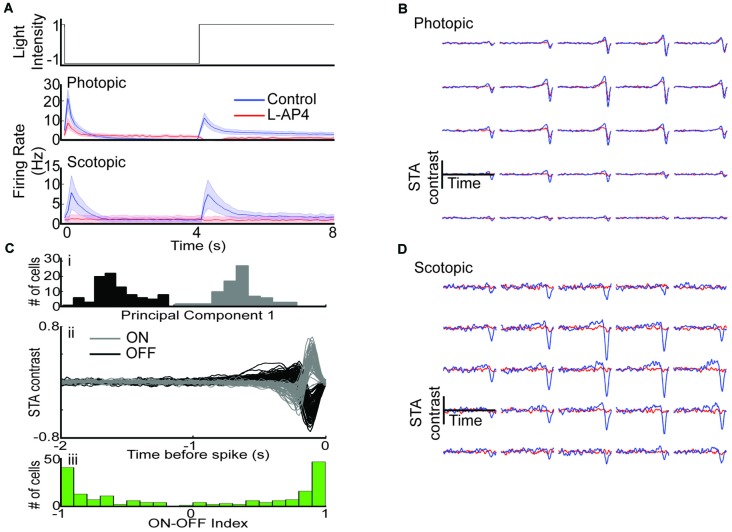
**Removal of photoreceptor to ON-BC signaling leaves photopic OFF RGC responsivity intact. (A)** Mean whole field response of all cells before (blue) and after (red) L-AP4 under photopic (row 2) and scotopic (row 3) conditions. ON responses are abolished while OFF responses are intact under photopic conditions while both ON and OFF responses are lost under scotopic conditions. **(B)** Example photopic space-time STA for a single cell before (blue) and after L-AP4 (red).** (Ci)** Scores from principal components analysis (PC1) were used to identify ON and OFF cells. **(ii)** STA waveforms from the two groups identified. **(iii)** ON-OFF index identified from whole field data. **(D)** Example scotopic space-time STA for a single cell before and after L-AP4.

The peak STA for each cell was concatenated and a PCA was performed to identify ON and OFF RGCs. Since STAs are inherently poor at distinguishing ON-OFF cells, we also used whole field stimulation to identify ON-OFF cells that may receive direct inputs from cone ON-BCs (Figure 2Ciii). While the OFF population defined by the STA contained OFF-dominated ON-OFF cells, excluding these cells had no meaningful effect on the results aside from decreasing sample size.

### Saturation-Threshold Nonlinearity

To calculate the saturation-threshold nonlinearity the recording segments were randomized and one half of the recording data was used to generate an STA. The other half of the data was convolved with the STA to determine the predicted generator potential. Predicted generator potential values were binned and the spike rates associated with the generator potential values within each bin were averaged. This allowed us to generate a plot comparing the predicted generator potential with cell firing rate. A normal cumulative distribution function (equation 2) was fit to this data (Chichilnisky, 2001).

(2) F(x) = αC(x,μ,σ)

Paramter α represents maximal firing rate while *C*( ) is the normal cumulative density function with parameters μ and σ, representing threshold and gain, respectively.

### Spatial Pooling and Surround Characterization

The STA was first fit to the product of a spatial Gaussian and the impulse response of a temporal filter (Chichilnisky and Kalmar, 2002). The spatial Gaussian determines the size of the 1-σ distance in the major and minor axis. This was used to determine identify 1-σ annular zones.

Temporal traces within zones 1–3 were combined to form a single center trace while those in zones 4–9 were combined to form a surround trace. By summing the first 150 ms of the center and surround trace we identified a single value to characterize the center and surround. The ratio of these values (surround/center) was calculated and is reported here as the Surround Polarity Index (SPI; Cowan et al., 2016b). A negative value indicates opposite polarity of the center and surround. A larger number indicates a stronger surround.

### The Sum of Separable Subfilters (SoSS) Model

The SoSS model is described in our previous report (Cowan et al., 2016b), but briefly it models the RF as the sum of up to five subfilters. Each subfilter has a unique temporal and spatial filter. The temporal filter is shown in equation 3 (Watson, 1986), and the spatial filter was a standard two-dimensional Gaussian. The product of these generates the space-time response for each subfilter *i*.

(3) $${f_{i}(t)\;=p_{i}(t/\tau_{i})}^{n_{i}-1}\left(e^{-\frac{t}{\tau_{i}}}\right)/\tau_{i}(n_{i}-1)!$$

The temporal properties (*τ*_i_ and *n*_i_) along with the scale (*p*_i_) were independent for each subfilter. All subfilters for a single cell had the same two-dimensional spatial Gaussian (center location and orientation), but its spatial extent was allowed to vary. We compared the annular-averaged raw data with annular-averages fit data with a weighted least squares regression. The weights were the square root of the number of spatial inputs in each annulus. For each cell an *F-test* was used for model comparison to determine how many subfilters were needed.

### Statistical Tests

Statistical tests and significance values are indicated in the text and methods. For comparison of populations we used the Student’s *t*-test when normally distributed, otherwise we used a Wilcoxon signed-rank test or Mann-Whitney *U* test. In all cases we applied a Bonferroni correction to account for multiple comparisons.

## Results

In order to determine the contributions of ON-BC mediated circuits to RGCs, we used a MEA to record from nine retinas before and after addition of L-AP4 (Figure 1A). Figure 1B shows a schematic diagram of the ON and OFF RGCs circuitry and the LAP-4 sensitive pathways are highlighted. We used whole field stimulation and white noise RF mapping to assay RGC light responses and spatiotemporal RFs under both conditions (Meister et al., 1994; Chichilnisky, 2001). Six retinas were stimulated at low photopic light levels, whereas three were stimulated at scotopic light levels.

### Removal of the ON-Bipolar Cell Light Responses Abolishes Scotopic but not Photopic Responsivity of OFF RGCs

We first determined the effect of L-AP4 on photopic light step responses across the RGC population (*n* = 215). The peak OFF response to the light step (as measured by maximum firing rate) was reduced, but the ON response was abolished (Figure 2A, row 2). In contrast, both ON and OFF responses were abolished across the RGC population under scotopic conditions (Figure 2A, row 3, *n* = 63). These results suggest photopic ON responses and scotopic ON/OFF responses require ON-BC mediated circuits.

We then mapped each cell’s RF by stimulating with a white noise checkerboard stimulus and using reverse correlation to generate a space-time spike triggered average (STA, see “Materials and Methods” Section). An example photopic STA before and after drug is shown in Figure 2B, while an example scotopic STA is shown in Figure 2D. A cell was considered responsive if any input within the STA exceeded 5 standard deviations from the mean and it had a firing rate > 0.25 Hz (see “Materials and Methods” Section). Under photopic conditions 100 of 171 cells were responsive with L-AP4, while under scotopic conditions only 2 of 22 cells were responsive with L-AP4.

In order to compare the effect of L-AP4 on photopic checkerboard responses for ON and OFF RGCs separately, we divided RGCs into ON (*n* = 79) and OFF (*n* = 92) groups based on their STA waveforms (Figures 2Ci,ii). Cells with ON-OFF responses to whole field light steps were retained for all comparisons in the article (see “Materials and Methods” Section). We find that 84 of 92 of OFF cells were responsive in the presence of L-AP4, while a much smaller fraction of ON cells were responsive. Furthermore, there was a significant correlation between a cell’s peak STA value before and after drug (*p* < 2E-5, Pearson’s correlation coefficient) indicating that the relative STA strength of each unit compared to others was preserved across conditions.

Overall, photopic OFF responses to whole field and checkerboard stimulation are preserved with L-AP4 allowing comparison of their properties before and after drug. Though responses were maintained, there is indication that response properties are altered (peak firing rate and peak STA contrast decrease, Figures 2A,B). This suggests that ON-BC mediated circuits contribute to OFF RGC photopic responses. In order to determine the role of these ON crossover pathways we compared response properties of OFF RGCs before and after drug in the following sections.

### ON Crossover Pathways Increase the Threshold of OFF RGCs

**Figure 3:**
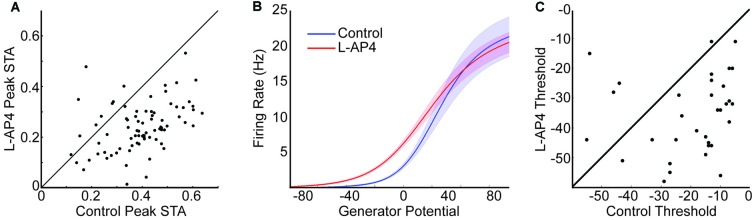
**ON Crossover pathways increase the threshold of OFF RGCs. (A)** Peak value in space-time STA compared before and after L-AP4. Unity line is shown in black, and most points lie below indicating larger peak STA in control conditions. **(B)** Mean static nonlinearity before (blue) and after (red) L-AP4 are shown. The shaded area depicts 1 standard error around each point. There is a significant leftward shift in L-AP4 which indicates a decrease in threshold. **(C)** The 5% threshold is compared before and after L-AP4. Most points fall below the unity line.

The peak STA is decreased in the example in Figure 2B. To determine if this was consistent across the population, we identified the peak STA value for each cell before and after L-AP4 and compared them (Figure 3A). Only OFF cells that were responsive before and after L-AP4 were compared (*n* = 84). Almost all cells fell below the unity line, indicating a significant decrease in peak STA with L-AP4. To further study the light response properties of the RGCs we calculated their static nonlinearity by comparing the predicted generator potential with spike rate and fitting the data with a normal cumulative distribution function (Chichilnisky, 2001; Della Santina et al., 2013, see “Materials and Methods” Section). This allowed us to parameterize each cell’s maximal spike rate, threshold and gain. The mean of the nonlinear fits under both conditions are shown in Figure 3B. Only cells that were well fit by the nonlinear model under both conditions were compared (*n* = 41). The gain and maximal firing rate was not significantly different with L-AP4. There was a significant decrease in threshold with L-AP4 (leftward shift for L-AP4 in Figure 3B, *p* < 1E-3). The scatter plot in Figure 3C shows that almost all cells had a lower threshold with L-AP4. In support of this result, we find an increased average firing rate in L-AP4 (3.7 vs. 5.6 Hz, *p* < 1E-3). In summary, we see that blocking ON crossover pathways significantly altered the response properties of OFF RGCs under photopic conditions. We hypothesized that some of these changes arise from alteration in inputs to the RGC. To test this hypothesis and determine which inputs are driven by the ON crossover pathways, the subsequent sections of this article study the RGC space-time filtering before and after drug.

### The Antagonistic Surround of OFF RGCs Is Mediated by Crossover Circuits

Linear RF studies have provided insight into space-time processing of the center, but the surround has often been ignored, partially due to inability to detect it (Kerschensteiner et al., 2008; Koehler et al., 2011). A recent study in mouse found that blockage of the ON crossover pathway did not alter the chromatic surround of JamB-RGCs (J-RGCs) which was proposed to be driven primarily by horizontal cells (Joesch and Meister, 2016). We wanted to determine if this was generally true for the achromatic surround of other OFF RGCs. To increase the number of cells for comparisons, the firing rate threshold was removed from the definition of responsive STA for subsequent comparisons. This increased the number of OFF RGCs with responsive STA under both conditions from 84 to 95.

**Figure 4:**
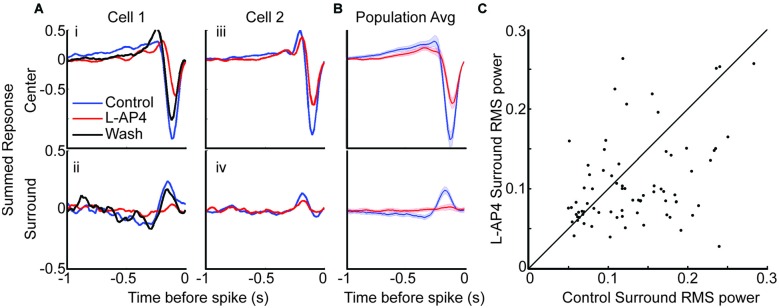
**The antagonistic surround relies heavily on contribution from crossover circuits. (A)** Space-time maps are divided into center and surround spatial regions. The temporal traces in the center region are combined (row 1) as are those in the surround region (row 2). **(i,ii)** An example cell with center and surround traces under control (blue), L-AP4 (red) and wash (black) conditions. This cell’s surround is lost with L-AP4. **(iii,iv)** An example cell that maintained surround response with L-AP4. **(B)** Mean center and surround before and after L-AP4 are shown. Shaded area indicates 1 standard error. **(C)** root mean square (RMS) power in the surround signal was calculated before and after L-AP4. There is a significant decrease in surround RMS power with addition of L-AP4. To account for noise contribution to the RMS power, we recalculated STAs to have the same number of spikes before and after L-AP4. Fewer cells had responsive STA under both conditions when STAs were recalculated with fewer spikes (*n* = 84).

We studied the antagonistic surround by dividing the space-time STA into distinct center and surround regions. The strength of each region was determined by combining the temporal traces of the STA that fell within it (see “Materials and Methods” Section). The center and surround traces from two cells are shown in Figure 4A. The cell in the first column had a significant linear antagonistic surround that disappeared in L-AP4, and returned after wash. The second column shows a cell that maintained a surround in the presence of L-AP4. The population average shows the surround response is lost in L-AP4, suggesting most cells are similar to cell 1 (Figure 4B). To quantify this effect, we calculated the root mean square (RMS) power for the surround trace of each cell before and after L-AP4. To ensure no difference in noise level between the two conditions, STAs were regenerated with the same number of spikes between conditions. There was a significant decrease in RMS power with addition of L-AP4 (Figure 4C). This indicates that the achromatic antagonistic surround of most OFF RGCs was altered by removing the ON crossover pathways.

### Crossover Circuitry Modulates the Temporal Profile of the Receptive Field Center of OFF RGCs

Crossover pathways have been shown to contribute to RGC center responses through feedback at BC inputs (Molnar and Werblin, 2007; Rosa et al., 2016) and feedforward directly to RGCs (Manookin et al., 2008). By using a recently developed method (Sum of Separable Subfilters (SoSS) model), we divide each RGC’s spatiotemporal response into multiple components with distinct spatial and temporal properties (Cowan et al., 2016b). Since these components are likely driven by distinct synaptic circuits, we wanted to determine which are driven by the ON crossover pathway.

**Figure 5:**
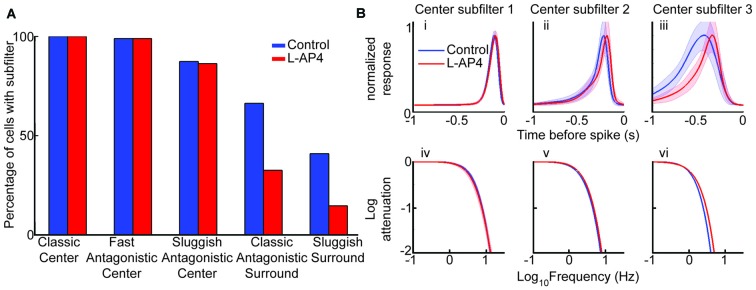
**The sluggish antagonistic center depends on crossover circuitry. (A)** The bar graph shows percent of cells requiring specified subfilter for model fit. There was no change in the presence of center subfilters with drug, but the presence of surround subfilter 1 (antagonistic surround) and 2 (center polarity surround) are decreased. **(B)** Average normalized impulse response for each center subfilter with 3 standard error highlighted by the shaded region **(i–iii)** and frequency spectrum for each subfilter **(iv–vi).** Significant difference only present in subfilter 3, with increased tuning after addition of L-AP4 **(iii,vi).** All comparisons are paired so only cells fit with the specific subfilter under both conditions are compared (*n* = 95, 94 and 83 for center subfilters 1, 2 and 3, respectively).

We performed the model fit for each cell before and after L-AP4 and quantified the presence or absence of components, which are referred to as subfilters (see “Materials and Methods” Section). There was no change in the presence of center subfilters, but surround subfilters were detected at a lower rate with L-AP4 (Figure 5A). The lower prevalence of surround subfilters corresponds to our earlier result showing a reduction of the antagonistic surround (Figure 3).

After seeing which subfilters are lost with L-AP4, we next looked to see how the drug altered the subfilters that remained, namely the triphasic RF center. The traces in Figure 5B show the mean response for each subfilter in a paired population. We found that the temporal tuning of center subfilters 1 and 2, which correspond to the commonly seen biphasic shape of linear STAs (Chichilnisky and Kalmar, 2002), was unaltered by L-AP4 (Figures 5Bi–iv). In contrast, the third center subfilter, which is sluggish and antagonistic, was significantly accelerated, as measured by its responses in time and frequency domains (Figures 5Bv,vi). These results suggest that the ON crossover pathway mediates the sluggish component of the center response.

### The Crossover Circuits Widen the Receptive Field Center of Most OFF RGCs

**Figure 6:**
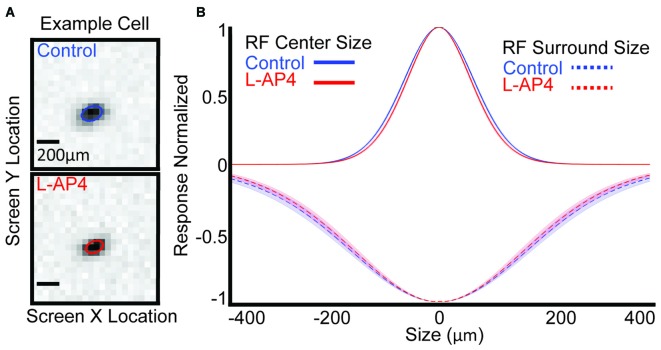
**Crossover circuitry widens the RF center. (A)** One example cell’s raw spatial data and spatial fit (colored ellipse) before **(blue)** and after L-AP4 **(red)**. **(B)** Population mean of center (solid line) and surround (dashed line) spatial extent is shown. Shaded area represents 1 standard error.

Since we saw that the ON crossover circuits play a role in shaping the temporal profile of the RF center, we wanted to see how they contribute to the size of the center. Figure 6A shows an example cell that decreases its RF center size with L-AP4. The RF center size for each cell is quantified by the size of center subfilter 1 and the mean across the population is decreased from 70.13 ± 1.12 μm (standard error) to 66.75 ± 1.30 μm with L-AP4 (Figure 6B, solid line, *p* < 5E-4, *t-test*). The other center subfilters also decrease in size with L-AP4. Center RF size is also significantly decreased if we use the standard quantification approaches (Chichilnisky and Kalmar, 2002; Della Santina et al., 2013). We also show the mean size of surround subfilter 1 across all cells which have a surround before and after L-AP4 (dotted line). There is not a significant change in size of the surround with L-AP4, but the number of cells containing a surround before and after drug is low (*n* = 16). We conclude that ON-BC crossover circuits widen the RF center size of most OFF RGCs.

## Discussion

In this article we found that ON crossover pathways provide significant inputs to the OFF RGCs, resulting in OFF center and ON surround response. These pathways are likely to be the major source of inputs under scotopic conditions since almost all light responses were lost in L-AP4. This also indicates that the OFF BC-mediated circuits are not strong enough to elicit scotopic light responses in OFF RGCs.

Under photopic conditions both the ON crossover and OFF BC pathways are active, because light responses are not lost when the ON crossover pathway is blocked. On the other hand, we found that application of L-AP4 significantly changed the spatial and temporal response properties of OFF RGCs under photopic conditions.

By using linear analysis, we found that the ON crossover circuitry widens the RF center and mediates the antagonistic surround. In addition, they provide sluggish ON response in the OFF RGC RF center, which prolongs its OFF response.

Center-surround antagonistic RFs and space-time processing are fundamental building blocks for visual processing and have been connected with ocular diseases such as glaucoma (Della Santina et al., 2013; Chen et al., 2015). By determining which pathways are providing these features we can find which retinal circuits could be damaged in disease states.

### ON Crossover Pathways Widen the Receptive Field Center of OFF RGCs

RGCs display a wide range of spatial tuning to help shape downstream visual encoding (Kolb, 1995). A classic example is that RGCs have decreased spatial tuning under scotopic light levels compared to photopic light levels (Dedek et al., 2008). This allows a larger collecting area to compensate for the fact that there are fewer photons. Most studies correlating this alteration in spatial tuning with retinal circuits describe the loss of antagonistic surround rather than a widening of the RF center (Cook and McReynolds, 1998; Sinclair et al., 2004; Dedek et al., 2008; Farrow et al., 2013). These studies found that removal of antagonistic surround widened spatial tuning and we see addition of L-AP4 removes the surround in most cells and decreases the center size. This would indicate that L-AP4 alters circuitry mediating surround strength and center size, whereas previous studies just removed the surround. We used white noise mapping and calculated the geometric mean, similar to other reports and our normal sizes are within their reported measurements (Della Santina et al., 2013). Other reports used different stimulation protocols (gratings, spots, etc) and different calculation methods (hypotenuse, area; Koehler et al., 2011).

RF center size relates to the dendritic field of RGCs and the spatial extent of their upstream inputs (Schwartz et al., 2012). Since L-AP4 is unlikely to alter the dendritic field size of RGCs, our results indicate that the inputs from ON crossover circuits driving the OFF RGC center responses have a wider spatial extent than the direct OFF-BC inputs. This would make sense given that amacrine cells would have more lateral convergence. It has been shown that OFF transient RGCs had decreased RF center size in a mouse model of glaucoma (Della Santina et al., 2013). It has also been suggested that the RBC-to-AII synapse is functionally altered in this model (Pang et al., 2015). It is possible that ON amacrine cells in the ON crossover pathways, such as AII amacrine cells, are altered in glaucoma, leading to a narrow RF center. These results would indicate that multiple narrow field amacrine cells shape the microstructure of the RF center. The population of cells we compare in this study represent a heterogeneous group of OFF cells. Though most cells decrease their RF size a subset seem unchanged. Utilizing pharmacology to dissect RGC subtypes would be fruitful for future studies. Identifying the role of distinct circuits, such as the ON crossover pathways mediating RF center size, helps us understand the normal function of these circuits and their roles in disease states.

### Multiple Synaptic Pathways Converge to Shape Temporal Filtering in the Receptive Field Center

As with spatial processing, upstream circuits also mediate temporal processing of the RGCs. It has been shown that dark adaptation broadens RGC temporal integration (Enroth-Cugell and Shapley, 1973; Pandarinath et al., 2010b). This could be accounted for by differences between the kinetics of rod and cone pathways, however investigators have shown that other circuits contribute to these shifts. For example, an increase in horizontal cell coupling via Cx57 is critical for slower tuning of RGCs under scotopic conditions (Pandarinath et al., 2010a). In studies of linear RFs, changes in temporal tuning are described as shifts of linear filters (Pandarinath et al., 2010a,b). These linear filters are the sum of many underlying features. The simplest example would be the combination of linear filters of inhibitory and excitatory inputs in the RGC dendrites (Joesch and Meister, 2016). In a previous study, we identified three linear filters with distinct temporal tuning in the RF center of almost all RGCs which were termed subfilters (Cowan et al., 2016b). Two of these subfilters, center subfilters 1 and 2, represent the classic biphasic temporal filtering present in RGCs (Chichilnisky and Kalmar, 2002) and BCs (Baccus and Meister, 2002). The biphasic shape provides bandpass frequency tuning, instead of the low pass tuning seen in monophasic filters. The addition of center subfilter 3, which is sluggish and antagonistic, gives the overall linear filter a triphasic shape. The speed of this third subfilter determines how fast the peak firing decays. A lower speed means the firing rate will decay slower, allowing the cell to respond to more sustained stimuli. Here we show that all three subfilters are present in L-AP4, but only center subfilter 3 speeds up its kinetics. This suggests the ON crossover pathway makes the RGC response more sluggish. In a recent report, zebrafish OFF BC inputs fell into three distinct temporal frequency ranges. The lowest frequency of these required ON BC mediated crossover circuitry (Rosa et al., 2016). These observations are consistent with our finding that the ON crossover pathways mediated the sluggish center subfilter 3 of OFF RGCs. It is possible that the same ON crossover pathway is responsible for mediating sluggish responses in both OFF BCs and OFF RGCs. Likely there are cone and rod ON BCs contributing to the crossover, and the loss of low frequency is removal of the rod BC component which has been shown to function under photopic conditions as well (Dedek et al., 2008; Ke et al., 2014).

### ON Crossover Pathways Mediate the Antagonistic Surround of Most OFF RGCs

Light of opposite polarity driving RGCs outside the center, known as the antagonistic surround response, is a basic feature of RGCs (Barlow, 1953; Kuffler, 1953). Center-surround antagonistic RFs are present at almost every level of the visual pathway, and are considered a basic building block for spatial information processing (Wu, 2010). In RGCs the antagonistic surround is mediated by horizontal cells in the outer retina (Werblin and Dowling, 1969; Mangel, 1991) and amacrine cells in the inner retina (Daw et al., 1990; Cook and McReynolds, 1998; Jacobs and Werblin, 1998). Amacrine cells can form ON or OFF circuits that signal to RGCs of the same or opposite polarity (Pang et al., 2007). Amacrine cell circuits signaling to RGCs of opposite polarity are crossover pathways (Werblin, 2010). Crossover pathways have been speculated to contribute to the antagonistic surround (Werblin, 2010). However, a recent study in the linear RF of mouse suggested that the surround of J-RGCs, a type of OFF cell, is not mediated by these crossover pathways (Joesch and Meister, 2016). In this report, we found a significant decrease in the antagonistic surround of most OFF RGCs when the ON crossover pathway was suppressed by L-AP4. Our results also support a subpopulation of cells having ON crossover independent surrounds.

## Author Contributions

JS participated in acquisition of data. JS and SMW contributed to conception of the work. JS, RLS, CSC and SMW contributed to analysis and interpretation. All authors contributed to drafting of the work and gave final approval for this version to be published. All agree to be accountable for all aspects of the work.

## Funding

National Eye Institute (NEI) RO1 #RO1EY004446 and #RO1EY019908. NEI T32 #T32EY007001. NEI F30 #F30EY025480. NEI Core #P30EY002520. Retina Research Foundation. Research to Prevent Blindness.

## Conflict of Interest Statement

The authors declare that the research was conducted in the absence of any commercial or financial relationships that could be construed as a potential conflict of interest.
